# Absence of norovirus contamination in shellfish harvested and commercialized in the Northeast coast of Brazil

**DOI:** 10.1590/1414-431X20209529

**Published:** 2020-09-18

**Authors:** K.M. Guarines, R.P.G. Mendes, M.T. Cordeiro, M.P. Miagostovich, L.H.V.G. Gil, K.Y. Green, L.J. Pena

**Affiliations:** 1 Departamento de Virologia, Instituto Aggeu Magalhães, Fundação Oswaldo Cruz, Recife, Pernambuco, Brasil; 2 Laboratório de Virologia Comparativa e Ambiental, Fundação Oswaldo Cruz, Rio de Janeiro, RJ, Brasil; 3 Caliciviruses Section, Laboratory of Infectious Diseases, National Institute of Allergy and Infectious Diseases, National Institutes of Health, Bethesda, MD, USA

**Keywords:** Norovirus, Real-time PCR, Crassostrea rhizophorae, Mytella guyanensis

## Abstract

Norovirus (NoV) is the main cause of gastroenteritis outbreaks worldwide. Although NoV spreads mainly from person to person, it is estimated that a large proportion of NoV outbreaks are caused by foodborne transmission. Bivalve mollusks are one of the most important foods involved in NoV transmission to humans. Little is known about NoV prevalence in shellfish harvested and commercialized in Brazil. The aim of this study was to map, for the first time, the distribution of NoV contamination in oysters and mussels harvested and commercialized in the coast of Pernambuco state, northeast Brazil. A total of 380 mollusks (260 oysters and 120 mussels) were collected between February and August 2017 either directly from harvesting areas or obtained from beach vendors at 17 sites in Pernambuco. Samples were processed and tested for NoV contamination using a SYBR Green real-time PCR assay. All samples were negative for NoV GI or GII contamination, suggesting a low risk of NoV contamination from this food source during the study period. Additional surveys in different areas of the Brazilian coast are warranted to monitor the risk of NoV infection upon seafood consumption.

## Introduction

Norovirus (NoV) is the main cause of gastroenteritis outbreaks worldwide (1). Every year, NoV infections result in nearly 685 million cases and 200,000 deaths, causing a significant economical impact (2,3). In Brazil, the virus has become the most detectable viral pathogen in acute gastroenteritis since the introduction of a rotavirus vaccination, with a detection rate from 4 to 50% (4,5).

NoV is a non-enveloped positive sense single-stranded RNA virus classified in the family Caliciviridae. Its genome is approximately 7.7 kb and encodes three open reading frames (ORF, 1–3) (6). The Norovirus genus includes seven genogroups (GI-GVII) that infect different species. Human norovirus (HuNoVs) belongs to genogroups GI, GII, and GIV (7). NoV can be transmitted by multiple routes, including person-to-person, through environment fomites, and contaminated water and food. The virus can persist for long periods in the environment and as few as 18 virus particles are able to establish an infection (8).

NoV transmission is most often mediated by direct human-to-human contact. However, it is estimated that 14% of NoV outbreaks in developed countries are caused by foodborne transmission, a rate could that can be higher in settings with poor hygiene and sanitation (9). Mollusks are one of the most important foods involved on NoV transmission. They are known for their bioaccumulation capacity since they filter the surrounding water for feeding (10). NoV is the principal agent detected in oyster-associated outbreaks of gastrointestinal disease (11-14).

The contamination of shellfish by NoV is influenced by numerous factors, including low solar radiation, low water temperature, low gage height, low salinity, heavy rainfall, and strong offshore wind. Among these variables, low water temperature is the most important indicator for oyster-associated NoV outbreaks (15). Viral detection in shellfish is important to evaluate water quality, which is usually based on bacterial parameters and not viral agents. This can mask the actual quality of water and food consumed by the population (16).

There is still much to be elucidated about the biology and ecology of this important foodborne pathogen (8). In Brazil, a few studies have detected HuNoV in shellfish harvested in South and Southeast Brazil (17-19). The Northeast region is one of the favorite travel destinations in Brazil because of its stunning coastline and rich seafood cuisine. One important state in the region is Pernambuco, where tourists and residents are usually attracted by its recreational waters and beach-related activities (20). One study found NoV prevalence in humans in Pernambuco to be 15% (21). To the best of our knowledge, no study has investigated the presence of NoV in shellfish in the Northeast region of the country. Thus, the aim of this work was to investigate the presence of NoV contamination in oysters and mussels harvested and commercialized in the coast of Pernambuco state, Brazil.

## Material and Methods

### Sampling

**Figure 1:**
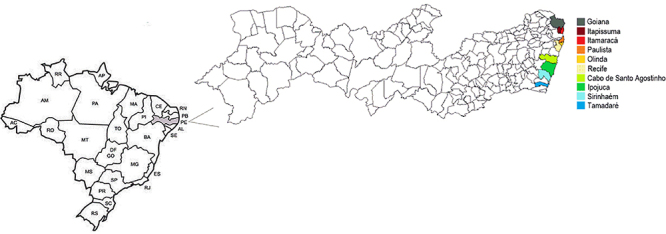
Study area. The map shows all states in Brazil and the gray-shaded area is the state of Pernambuco, where the study was carried out. Colored areas indicate the counties visited for shellfish collection.

A total of 380 mollusks, 260 mangrove oysters (*Crassostrea rhizophorae*) and 120 mangrove mussels (*Mytella guyanensis*), were collected between February and August 2017 for this study. They were acquired *in natura* directly from harvesting areas (n=250) or obtained from beach vendors (n=110). In some cases, samples were harvested directly from mangrove by the team members (n=20). A total of 17 sites were visited in the following coastal cities in Pernambuco state, Brazil: Goiana (Atapuz, Barra de Catuama, and Tejucopapo beaches), Itapissuma (Itapissuma beach), Itamaracá (Itamaracá and Pilar beaches), Sirinhaém (Barra de Sirinhaém beach), Recife (Boa Viagem, Brasília Teimosa, and Ilha de Deus beaches), Cabo de Santo Agostinho (Gaibu beach), Paulista (Maria Farinha and Pau Amarelo beaches), Ipojuca (Porto de Galinhas beach), Tamandaré (Tamandaré beach) (Figure 1).

Mollusks were transported under refrigeration, using chiller blocks, to the Virology Department of the Aggeu Magalhães Institute (Fiocruz). During collection, water temperature, ambient air temperature, and source of the water consumed by the population were recorded. For each site, 10 oysters and/or 20 mussels were collected.

### Shellfish processing

Mollusks processing was performed as described in a previous study (22). First, the shellfish were washed with running water and then shucked individually using a knife. Their digestive tissues were dissected using a sterile scalpel and transferred to Petri dishes, where they were finely chopped. Samples from the same site were weighed and pooled to form portions of 1-2 g. After, the same volume of proteinase K at 100 µg/mL was added, samples were mixed and incubated for 60 min at 37°C in a shaker incubator with agitation at 320 rpm. Samples were then incubated at 65°C during 15 min in a water bath to inactivate proteinase K. Finally, samples were centrifuged at 3000 *g* for 5 min at 4^o^C. Supernatant was kept under 4°C until RNA extraction.

### RNA extraction

Viral RNA was extracted from 140 µL of processed shellfish supernatant using QIAmp^®^ Viral RNA Mini kit (Qiagen, USA), according to the manufacturer's recommendations. RNA was eluted using 60 µL of AVE buffer and stored at -80°C until use.

### NoV positive controls

Positive NoV GI and GII controls (fecal suspension) were provided by Dr. Marize Miagostovich (Fundação Oswaldo Cruz-FIOCRUZ/RJ) and the Pernambuco Central Laboratory of Public Health (LACEN/PE), respectively. Controls were cloned, transcribed, and used in all real-time RT-PCR reactions.

Specific fragments of NoV GI and GII were amplified by conventional RT-PCR using ImProm-II™ Reverse Transcription system (Promega, USA) and GoTaq^®^ Green Master mix (Promega) following the manufacturer's instructions. We used previously designed primers (22,23) that target the conserved region at the ORF1-ORF2 junction of the genome (Table 1). Amplicons were purified with the Illustra GFX PCR DNA and Gel Band Purification kit (GE Healthcare Life Sciences, USA) and cloned into the pGEM^®^-T Easy vector (Promega) using 1:3 or 1:8 vector:insert molar ratios. NoV positive clones were confirmed by colony PCR using the same primers used for cloning and by Sanger sequencing. In brief, purified amplicons were sequenced using the same primers used for cloning and the BigDye Terminator v3.1 Cycle Sequencing kit (Applied Biosystems, USA). Sequencing was performed on an ABI Prism 3100 Capillary Automatic DNA analyzer. Sequences were analyzed using Bioedit software, v7.0.5 (USA) and submitted to Blast analysis (http://www.ncbi.nlm.nih.gov/blast/Blast.cgi) to confirm NoV identity.

**Table 1:** Primers used in the study.

Primer	Sequence	Reference
JJV1F	GCC ATG TTC CGI TGG ATG	(22)
JJV1R	TCC TTA GAC GCC ATC ATC AT	(22)
JJV2F	CAA GAG TCA ATG TTT AGG TGG ATG AG	(22)
COG2R	TCG ACG CCA TCT TCA TTC ACA	(23)

JJV1F and JJV1R primers amplify norovirus (NoV) GI strains while JJV2F and COG2R primers are specific for NoV GII.

*In vitro* transcription was carried out using SacII-linearized plasmids and the MEGAscript^®^ SP6 Transcription kit (Ambion, Life Technologies Corporation, USA), following the manufacturer's recommendations. Briefly, the digested product was purified using Illustra GFX PCR DNA and Gel Band Purification kit (GE Healthcare Life Sciences). Next, transcription reactions were performed with 8 µL of linearized plasmid DNA, 2 µL of 10× SP6 buffer, 2 µL of each phosphate ribonucleotide (ATP, GTP, CTP, and UTP), and 2 µL of SP6 enzyme. RNA was treated with 5 µL of TURBO™ DNase (Ambion, Life Technologies Corporation) per reaction and incubated at 37°C for 30 min. Transcribed RNAs were treated with 115 µL of nuclease-free water and 15 µL of ammonia acetate. RNA was precipitated with 310 µL of ethanol, and then resuspended and eluted in 36 µL of nuclease-free water and 4 µL of RNaseOut^®^ (Invitrogen, USA).

To determine the analytical sensitivity of real-time RT-PCR, serial 10-fold dilutions (from 10^1^ to 10^11^ copies/µL) of an *in vitro*-transcribed NoV GI or GII RNA were made in nuclease-free water and used for the assays. All experiments were done in duplicate. RNA copy number was calculated based on the formula:

$$\frac{\text{RNA concentration}\;(\text{g}/µ\text{L})}{\text{Transcript size}\;(\text{bp})\;\times \;340}\;\times \;6,022\;\times \;10^{23}$$

The standard curve was also used for viral quantification in unknown samples.

### Oyster spike experiments

Oysters were artificially contaminated with serial 10-fold dilutions of a NoV positive sample to analyze the sensitivity of the real-time RT-PCR assay. This experiment also allowed determining the absence of real-time RT-PCR inhibitors in oyster samples. Briefly, one gram of dissected oyster diverticulum was spiked with 100 µL of serially diluted (undiluted to 1:10,000 dilution) NoV GII-positive fecal suspension (2.7×10^4^ copies/µL) and then processed as described above.

### NoV molecular detection

Real-time RT-PCR was performed using the GoTaq^®^ 1-Step RT-qPCR system (Promega). The primers, which had been designed in previous studies (22,23), are described in Table 1. The 20 µL reaction was carried out using 10 µL of GoTaq^®^ qPCR Master Mix 2X, 6 µM of each specific primer, 0.18 µL of CXR reference dye, 0.4 µL of GoScript^®^ RT mix, 6.22 µL of nuclease free water, and 2 µL of RNA. We performed NoV GI and NoV GII amplifications separately. PCR conditions were as follows: RT at 50°C for 30 min; initial denaturation at 95°C for 10 min; 40 cycles of 95°C for 10 s, 60°C for 30 s and 72°C for 30 s, finishing with melt curve. Positive (standard curve) and non-template (nuclease-free water) controls were used in every experiment. All PCR reactions were run in duplicate. Melt curve analysis was carried out using the Applied Biosystems 7500 Software v2.0.6 (Life Technologies, USA).

### Statistical analysis

All statistical analyses were performed using GraphPad Prism version 5.01 for Windows (GraphPad Software, USA) and IBM SPSS Statistics 22.0 (USA) softwares. Statistically significant differences were defined as P<0.05.

## Results

### Shellfish sampling

**Figure 2:**
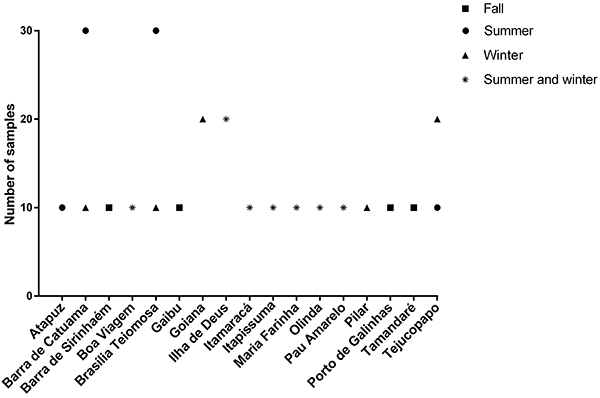
Beach names, number of samples, and seasons of sample collection.

A total of 380 mollusks (260 oysters and 120 mussels) were collected. Beach names, number of samples collected, and seasons when sites were visited are reported in Figure 2. All shellfish were processed and the digestive tissues of samples from the same site were pooled to form portions of 1-2 g. At the end, 233 tissue samples were obtained. RNA was extracted from all these samples and kept at -80°C until real-time RT-PCR.

### Sample characterization

**Figure 3:**
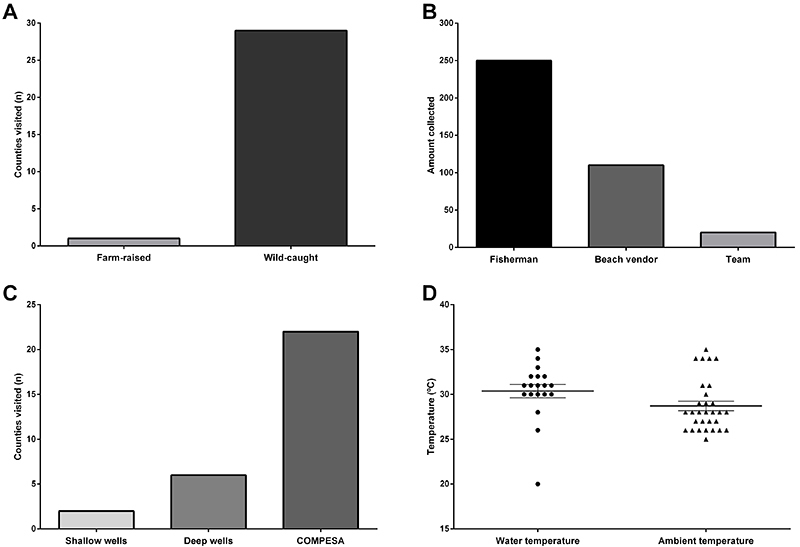
. Sample characteristics. **A**, Source of shellfish. **B**, Origin of samples obtained. **C**, Source of water consumed by the population. **D**, Water and ambient temperature at time of collection. COMPESA: community water system company.

The majority of mollusks collected for this study were wild-caught (96.7%) and only a small fraction was farm-raised (Figure 3A). Most shellfish samples were obtained from fishermen (n=250), followed by beach vendors (n=110), and the research team (n=20) (Figure 3B). The main source of water consumed by the population in the cities visited was provided by Compesa (73.3%), a community water system company responsible for water supply and sanitation services in Pernambuco state. In addition, 20% of drinking water was provided from deep well sources and 6.7% from shallow wells (Figure 3C).

During visits, water and ambient temperatures were also measured and recorded. The mean ambient temperature was 28.7°C (25-35±2.092°C). Average water temperature was 30.37°C (20-35±3.20°C) (Figure 3D).

### One-step SYBR^®^ Green real-time RT-PCR standardization

In order to detect NoV contamination in shellfish, we first developed a one-step SYBR^®^ Green real-time RT-PCR assay using previously designed primers (22,23). Positive NoV GI and GII controls were amplified from human fecal suspensions and cloned into a plasmid. Successful cloning of desired inserts was confirmed by colony PCR and Sanger sequencing of plasmids (data not shown). NoV RNA was produced by *in vitro* transcription of plasmids and quantified by spectrometry. RNA concentration obtained was 4.85×10^12^ ssRNA copies/µL for GI and 4.79×10^12^ ssRNA copies/µL for GII.

**Figure 4:**
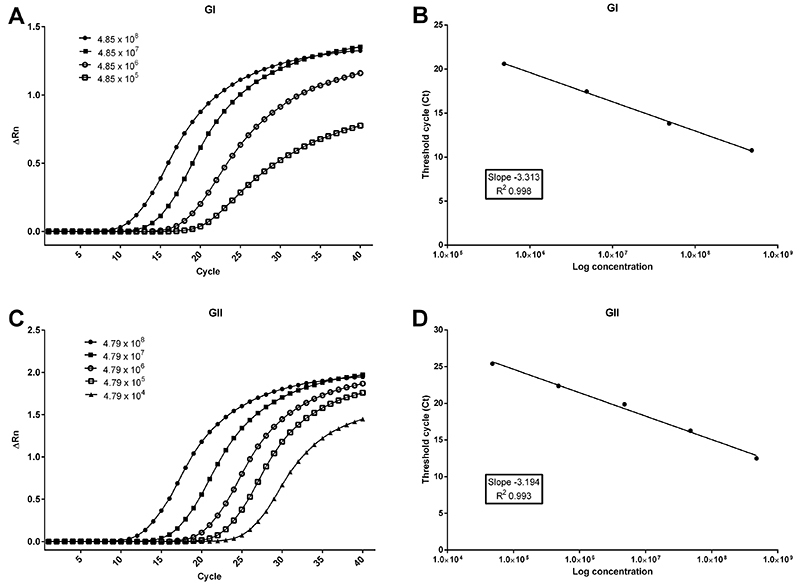
One-step SYBR^®^ Green real-time RT-PCR assay for norovirus (NoV) GI (**A** and **B**) and GII (**C** and **D**). The linearity of the assay was determined using a serially 10-fold diluted NoV RNA transcribed from plasmids containing either the NoV GI (**A**) or GII (**C**) targeted region. **B** and **D**, Standard curves generated from amplification of NoV GI and GII by real-time RT-PCR.

Serial 10-fold dilution of transcribed NoV RNA was used to construct standard curves using concentrations varying from 10^1^ to 10^8^ copies/µL. The detection limit of the real-time RT-PCR assay was defined as 4.85×10^4^ ssRNA copies/µL for NoV GI and 4.79×10^2^ ssRNA copies/µL for NoV GII. However, linearity of RNA amplification was obtained using 4.85×10^5^-4.85×10^8^ copies for GI and 4.79×10^4^-4.79×10^8^ copies for GII, with efficiency of 100 and 105%, respectively (Figure 4).

To determine the assay sensitivity and analyze the processing protocol, a spike curve in oysters was constructed using a positive stool sample. Approximately 2.7×10^4^ ssRNA copies/µL of NoV GII RNA was used to perform the spike experiment. This sample was diluted until 2.7 copies/µL created a 10-fold dilution curve. Each dilution was inoculated into 1-g portions of oyster digestive tissue and samples were processed for RNA extraction. Under this condition, the real-time RT-PCR was capable to detect NoV up to 2.7×10^3^ copies/µL concentration point.

### Analysis of shellfish samples for NoV contamination

**Figure 5:**
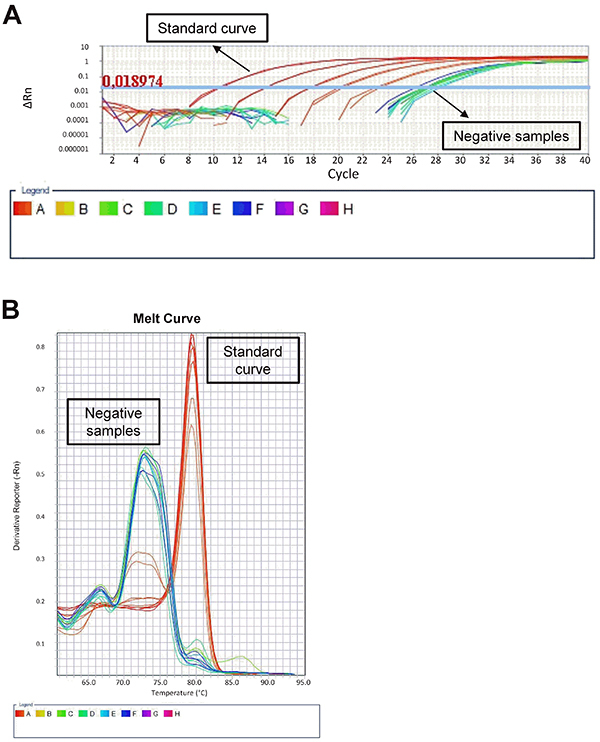
Analysis of shellfish samples for norovirus (NoV) contamination. **A**, Shellfish samples were processed and tested for NoV contamination by real-time RT-PCR along with positive and negative controls. **B**, Melt curve analysis to discriminate between NoV positive and negative samples. A-H: positions of samples on PCR plate.

All 233 samples were tested in duplicate for NoV GI and NoV GII, together with NoV transcribed RNA (standard curve) and non-template controls, by the one-step SYBR^®^ Green real-time RT-PCR assay. None of the shellfish samples had detectable NoV contamination (Figure 5A).

Melt curve analysis was done to discriminate between the negative (water controls and negative shellfish samples) and positive NoV. Amplification of NoV GII positive controls produced amplicons with a melting temperature 7°C higher than negative samples (80 *vs* 73°C), which allowed a clear differentiation between positive and negative samples (Figure 5B). Amplification of NoV GI controls produced a similar melt curve pattern (data not shown).

## Discussion

Information about NoV prevalence in shellfish harvested and commercialized in Brazil is scarce and incomplete. This study was the first survey of the occurrence of NoV in bivalve mollusks harvested and commercialized in the coast of Pernambuco state, northeast Brazil.

One major obstacle in the laboratory diagnosis of NoV infection has been the lack of an efficient tissue culture system for isolating viruses directly from field samples (24). Thus, RT-PCR has been increasingly used for NoV detection around the world (22,23,25,26). The development of new techniques for rapid detection and molecular characterization of NoV is highly desirable for better understanding of NoV epidemiology (27). In this study, we standardized a rapid and inexpensive diagnostic tool for NoV detection in shellfish based on real-time RT-PCR with SYBR Green technology. Positive RNA controls for RT-PCR were produced by *in vitro* transcription of cloned cDNA, a method that allows production of RNA controls in large quantities for diagnostic purposes. The use of RNA standard curves also enables viral load determination in chosen samples (25).

Despite being a cheaper alternative, SYBR Green sensitivity is usually lower compared to TaqMan^®^ or methods involving the use of probes (28). In our spike experiment, we showed a sensitivity of 2.7×10^3^ RNA copies/µL for real-time RT-PCR and proved that there was no significant influence of oyster's inhibitors on virus detection. Standard RNA curve could detect 4.85×10^4^ copies of NoV GI and 4.79×10^2^ copies of NoV GII. This sensitivity was higher than the one described by Scipioni and co-workers (29), who obtained linearity in NoV amplification in human stool samples in the range of 5.8×10^6^ to 5.8×10^11^ copies. Similar results were also obtained by other authors (30), who developed a SYBR Green-based real-time RT-PCR for NoV detection in lettuce, cherry tomatoes, and green onions. Recently, Oshiki and co-workers developed a microfluidic nested PCR amplification method for NoV detection in oysters and found that the assay could detect 10^2^-10^5^ copies/g digestive tissue (31). Thus, our one-step SYBR^®^ Green real-time RT-PCR assay showed potential for routine use in diagnostics and monitoring of NoV contamination in mollusks.

Bivalve mollusks are an important source of NoV contamination and have been linked to several outbreaks in humans (10,13). Scientists worldwide have done NoV survey studies in mollusks, but the majority of investigations have been undertaken in countries with a temperate climate (22,31-35). These studies have obtained a variable detection rate. For example, Torok et al. (36) conducted a national prevalence survey for NoV in a total of 300 oysters harvested in Australia and did not find any positive samples. Low prevalence of NoV in shellfish or even absence of contamination has also been reported in other countries such as Greece, India, Japan, The Netherlands, Norway, and Spain (13). On the other hand, high prevalence of NoV contamination has also been reported. For instance, Le Mennec et al. (33) detected 65% NoV positivity in oysters from a production area repeatedly implicated in oyster-related gastroenteritis in France. It is hard to compare different studies because there are variables such as the use of distinct processing and detection methods, virus stability, water temperature, and season that differ among studies (36,37).

In Brazil, only a few groups had investigated NoV contamination in shellfish in the South and Southeast regions of the country. The detection rate in these studies has varied from 0 to 4.8% (17-19). This study was the first to investigate NoV contamination in shellfish from the Northeast region of the country. All 380 oysters and mussels samples collected were negative for NoV GII and GI, suggesting a very low prevalence of NoV in these shellfish. This was in agreement with Souza et al. (17), who did not detect any NoV GI or NoV GII positive samples in oysters harvested in regular cultivation areas in South Brazil. Our results are also in accordance with those described by Souza et al. (19). They collected samples of *Anomalocardia brasiliana* clams monthly between November 2014 and April 2016 in South Brazil and found only two positive samples for NoV GI and none for NoV GII. Keller et al. (18) studied bacterial and enterovirus contamination in shellfish harvested in an area impacted by a continuous discharge of domestic sewage in Southeast Brazil. Although they detected a high prevalence of adenovirus and rotavirus contamination in both water and mussel samples, NoV was found in only 4.8% of water samples and was not detected in the mussels.

It is important to analyze all factors involved in mollusk contamination, improving epidemiological surveillance data. Detailed information of these elements could help measure contamination risk and prevent foodborne transmission (27). Oyster-associated NoV outbreaks are influenced by the sum of different factors, such as low solar radiation, low water temperature, low gage height, low salinity, heavy rainfall, and strong offshore wind (15).

Often, NoV outbreaks occur in winter and cold months (38,39). Low temperature is a prime factor for viral prevalence and viral load in oysters. Higher viral concentrations were found in oysters collected in waters with a temperature under 5°C than in oysters collected in areas with temperature above 10°C. The processes of bioaccumulation and purification of oysters may also be compromised due to the decrease of its metabolism associated with low temperatures (40).

To determine the possible seasonal variation of NoV prevalence, we visited collection areas in different months. However, Northeast Brazil is a warm region, with high incidence of solar radiation almost year-round and the seasons are not well defined. The mean temperature of water and ambient in our study were 30.37 and 28.7°C, respectively. Chenar and Deng (15) predicted that high temperatures and high solar radiation were the two major factors that reduce the risk of NoV contamination. High solar radiation increases temperatures and UV rays incidence, promoting NoV inactivation and decreasing its stability. The high water temperature associated with oyster harvest for human consumption in suitable catchment areas in Pernambuco may explain the absence of detectable NoV contamination in our study.

### Conclusions

Monitoring risks of NoV infections is extremely important to prevent outbreaks among shellfish consumers and contribute to improvement of the estuarine environment. Our results suggested that the food safety risk related to NoV acquisition from shellfish harvested and commercialized in Pernambuco was low for the study period. Results of the current survey provide important information for the gastronomic tourism in Pernambuco and for the shellfish production chain in Northeast Brazil. Additional studies in other coastal states in Brazil are warranted to determine the prevalence of NoV in bivalve mollusks and will contribute to the understanding of the ecoepidemiology of NoV infection in tropical countries.
